# Health Insurance Coverage Among Same-Sex vs Different-Sex Couples

**DOI:** 10.1001/jamanetworkopen.2025.32844

**Published:** 2025-09-19

**Authors:** Benjamin J. Harrell, Nicole E. Jones, Gabe H. Miller

**Affiliations:** 1 Department of Economics, Trinity University, San Antonio, Texas; 2 Department of Sociology and Anthropology, Trinity University, San Antonio, Texas; 3 Department of Sociology, University of Alabama at Birmingham, Birmingham; 4 Center for the Study of Sexual and Gender Health, University of Alabama at Birmingham, Birmingham

## Abstract

**Importance:**

Recent research suggests that since the Patient Protection and Affordable Care Act and the US Supreme Court’s ruling in *Obergefell v Hodges,* lesbian, gay, bisexual, and/or transgender (LGBT) individuals are now at or beyond parity in insurance coverage with their non-LGBT counterparts, a reversal of a long-standing health disparity, but shows that gains have not been equitable.

**Objective:**

To examine health insurance coverage of same-sex and different-sex couples by marital status, sex, insurance provider, state of residence, and income distribution.

**Design, Setting, and Participants:**

This cross-sectional study was a secondary data analysis of 2008 to 2022 American Community Survey (ACS) data. The ACS is an annual, nationally representative survey of over 3 million households in the US, and study participants included adults aged 18 to 64 years in a cohabitating relationship. Data were analyzed in October and November of 2024.

**Exposure:**

Cohabitation in a same-sex or different-sex couple.

**Main Outcomes and Measures:**

The primary outcome was reporting any health insurance coverage in the 2008 to 2022 ACS results. A series of linear probability models was estimated to assess the association between same-sex couple status and the probability of being covered by any health insurance.

**Results:**

The sample included a total of 20 938 414 respondents (mean [SD] age, 45.57 [11.86] years). The majority of respondents in the study sample were women (11 139 506 participants; 53.2% unweighted; 52.6% weighted), were married (13 988 414 participants; 63.2% unweighted; 67.0% weighted), had less than a bachelor’s degree (13 408 218 participants; 65.1% unweighted; 64.1% weighted), and were employed (15 921 280 participants; 76.5% unweighted; 76.0% weighted). Participants reported a median (IQR) household income of $88 000 ($84 000). Respondents had a high level of insurance coverage (18 587 258 participants; 88.0% unweighted; 89.0% weighted) with 3 453 491 participants (16.8% unweighted; 16.5% weighted) reporting public and 14 248 083 (65.8% unweighted; 67.4% weighted) reporting private coverage. In further heterogeneity analyses, respondents in same-sex couples were more likely to be insured by employer-sponsored insurance than their counterparts in different-sex couples and less likely to be insured by all other insurance types, including public health insurance. Respondents in same-sex couples in the bottom quintiles of the income distribution were less likely to be insured compared with their counterparts in upper quintiles. Finally, respondents in same-sex couples living in states that had not yet expanded Medicaid (Alabama, Georgia, Kansas, Mississippi, South Carolina, Tennessee, and Wyoming) were less likely to be covered across the sample period.

**Conclusions and Relevance:**

This cross-sectional study examining insurance coverage among same-sex vs different-sex couples documented gains in health insurance status among individuals in same-sex couples in the past decade in the US. These gains, mostly realized by individuals of high socioeconomic status, reversed one of the defining health disparities for sexual minority adults of the late 20th century, but more must be done to ensure that these gains are distributed across the income distribution, especially to those living in states that have not yet expanded access to public health insurance.

## Key Points

**Question:**

Is health insurance coverage different for same-sex vs different-sex couples?

**Findings:**

This cross-sectional study of 2008 to 2022 American Community Survey (ACS) data for 20 938 414 individuals found that recent gains in insurance coverage among same-sex couples have largely been concentrated among key groups: male couples, married couples, those insured by plans offered by their employers, those in states that have expanded Medicaid, and those at the top of the income distribution.

**Meaning:**

These findings suggest that although same-sex couples now report, on average, higher rates of insurance coverage than different-sex couples, more must be done to ensure that these gains are distributed equitably, especially to those living in states that have not yet expanded access to public health insurance.

## Teaser

This cross-sectional study compares health insurance coverage rates between same-sex and different-sex couples in the US from 2008 to 2022 by marital status, type of insurer, state of residence, and household income.

## Introduction

Nationwide legalization of same-sex marriage and key provisions of the Patient Protection and Affordable Care Act (ACA) have narrowed the gap in insurance coverage for sexual and gender minority (SGM) adults.^1,2,3,4^ This narrowing is historic. Before *Obergefell v Hodges *(hereafter, *Obergefell*) and *United States v Windsor *(hereafter, *Windsor*), many individuals in same-sex marriages were prevented from sharing employer-sponsored insurance with their spouse.^5^ While some states offered mechanisms by which same-sex couples could share coverage with same-sex domestic partners, employer offerings of those benefits declined sharply immediately following *Obergefell*.^6^

Moreover, SGM adults have and continue to experience unmet medical needs, before the ACA to present,^4,7^ often attributed to a constellation of factors (eg, additional out-of-pocket costs, practitioners’ lack of competency of lesbian, gay, bisexual, transgender [LGBT] health, and discrimination).^7,8,9^ Recent work has found improvements in insurance coverage among low-income same-sex couples following the enactment of ACA Medicaid expansion^3^ and that gay and/or lesbian married and cohabiting adults are more likely to have private insurance compared with their single counterparts.^10^ However, adults in same-sex couples are still experiencing disparities in employer-sponsored insurance, particularly in the South and Midwest.^11^ Despite this, there is a paucity of research that explores the contours of variation in insurance coverage by sexual orientation and other sociodemographic characteristics.

We build on previous work using pooled 2008 to 2022 American Community Survey (ACS) data. In this article, we use the largest sample of publicly available data on SGM adults, enabling more granular analyses to investigate 3 hypotheses. First, we hypothesize that previously documented improvements in insurance coverage among same-sex couples are largely attributable to those in marriages. Second, we hypothesize that these improvements vary by state context. Third, we hypothesize that these gains are concentrated at the top of the income distribution.

## Methods

### Study Design

In this cross-sectional study, we performed a secondary data analysis of 2008 to 2022 ACS data.^12^ The University of Alabama at Birmingham institutional review board designates ACS data as not constituting human participants research; therefore, the study was exempt from institutional review board approval and informed consent requirements in accordance with 45 CFR §46. This investigation is reported using Strengthening the Reporting of Observational Studies in Epidemiology (STROBE) reporting guidelines for cross-sectional studies.

### Study Population and Data Source

Data for this study are drawn from 2008 to 2022 ACS data, an annual, nationally representative survey of over 3 million households in the US. Data were downloaded from the Integrated Public Use Microdata Series (IPUMS-USA) at the University of Minnesota Population Center.^12^ The ACS did not directly collect data on sexual orientation during our sample window; however, it is possible to identify same-sex couples using a variety of methods. We use the stated relationship of the respondent to the head of household.^3^ While none of these methods allow for direct observation of sexual orientation, the ACS remains the largest publicly available sample of SGM adults in the US, allowing for a variety of stratifications not available using other data products.

We eliminated from our sample anyone who is single or older than 64 years, since we expect the latter to be covered, at least in part, by Medicare. In analysis including coverage type, which includes Medicare, we relax this restriction.

### Outcomes, Exposures, and Covariates

We examined whether the ACS respondent reported any health insurance coverage, with the primary exposure of interest being whether the respondent is in a same-sex or different-sex couple. Covariates examined include sex (male or female), race and ethnicity (Hispanic, non-Hispanic Asian, non-Hispanic Black, non-Hispanic White, and other race, which referred to American Indian or Alaska Native, multiracial, other, or unknown), age (18-64 years), marital status (married or unmarried), educational attainment (less than a bachelor’s degree or bachelor’s degree or higher), earned income, and employment status (employed or unemployed).

### Statistical Analysis

#### Primary Analyses

All analyses were conducted in October and November of 2024 using Stata statistical software version 18 (StataCorp).^13^ Descriptive statistics were calculated and are presented for variables included in models. We next plotted unadjusted trends with 95% CIs of insurance rates among individuals in same-sex couples and different-sex couples from 2008 to 2022 and then restricted our analysis to 2013 to 2022 to illustrate the evolution of insurance status trends overall and by marital status. We further disaggregated these trends by sex and present trends for men vs women from 2013 to 2022. Finally, we estimated a series of linear probability models assessing the association between same-sex couple status and the probability of being covered by any health insurance. We began by estimating a model without demographic controls, then iteratively added covariates, state and year fixed effects, and finally estimated a model that fully interacts same-sex couple status, marital status, and sex. All analyses used 95% CIs, and 2-sided *P* values are reported with a *P* < .05 threshold for significance.

#### Secondary Analyses

To further explore how insurance rates have evolved for same-sex and different-sex couples over time, we performed a series of heterogeneity analyses. Given that state policy environments (including the decision to expand eligibility for Medicaid) affect insurance rates, we began by plotting insurance rates for same-sex and different-sex couples over time, state by state, including the District of Columbia. Similarly, we disaggregated coverage among same-sex and different-sex couples by insurance type: insurance provided by one’s employer, privately purchased insurance, Medicare, Medicaid, and other types of public health insurance (provided by TRICARE, the US Department of Veterans Affairs, or the Indian Health Service). Finally, since marriage is positively associated with socioeconomic status and human capital accumulation, we plotted insurance rates for same-sex and different-sex couples across their respective income distributions to analyze how differences in insurance status might be associated with household income.

## Results

We present descriptive statistics in the eTable in Supplement 1. The sample included a total of 20 938 414 respondents (mean [SD] age, 45.57 [11.86] years). The majority of respondents in the study sample were women (11 139 506 participants; 53.2% unweighted; 52.6% weighted), were married (13 988 414 participants; 63.2% unweighted; 67.0% weighted), had less than a bachelor’s degree (13 408 218 participants; 65.1% unweighted; 64.1% weighted), were employed (15 921 280 participants; 76.5% unweighted; 76% weighted), and reported a mean (SD) annual personal income of $43 944.33 ($61 608.05). Respondents had a high level of insurance coverage (18 587 258 participants; 88.0% unweighted; 89.0% weighted) with 3 453 491 participants (16.8% unweighted; 16.5% weighted) reporting public and 14 248 083 participants (65.8% unweighted; 67.4% weighted) reporting private coverage. Descriptive analysis comparing individuals in different-sex and same-sex couples is shown in the eTable in Supplement 1. Insurance coverage significantly varied by couple type across the sample period, with individuals in different-sex couples having a slightly lower prevalence of any health insurance coverage vs same-sex couples (18 382 787 respondents [89.0%] vs 204 471 respondents [91.0%]; χ^2^ = 801.3; *P* < .001).

**Figure 1.**
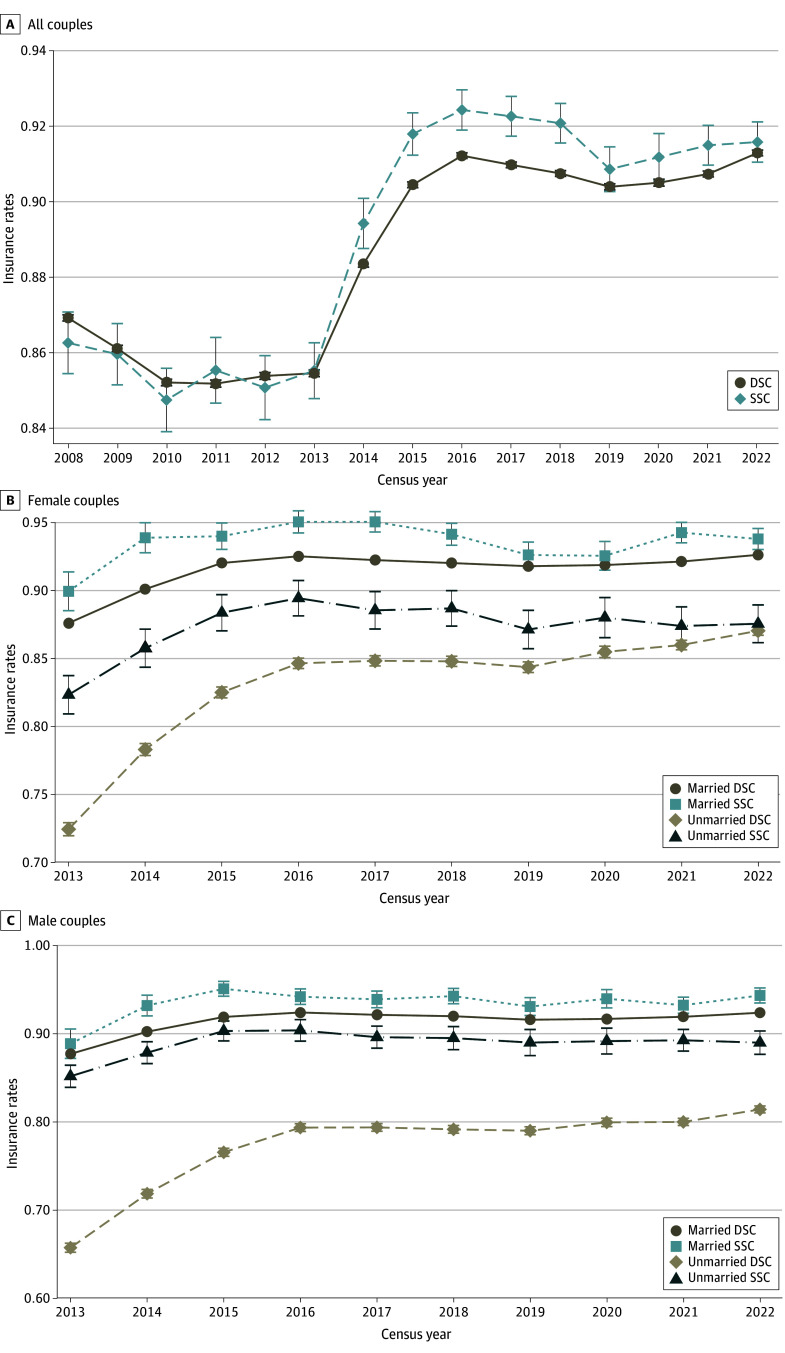
Insurance Rates Among Same-Sex Couples (SSCs) and Different-Sex Couples (DSCs), Overall and by Sex, 2008 to 2022 Error bars denote 95% CIs.

Figure 1 presents trends of insurance coverage among individuals in same-sex and different-sex couples from 2008 to 2022. Our primary analysis shows that by 2008, individuals in same-sex couples were already reaching parity in health insurance coverage compared with those in different-sex couples, despite small (but not statistically significant) penalties to coverage in many years. The most striking change in this trend occurred following 2013, when for the first time the historical disparity between these 2 groups reversed, peaked in 2018, and converged in the most recent years of available data, 2022. After *Windsor*, a clear marital benefit emerged, with individuals in married same-sex couples, particularly male couples, reporting the highest rates of health insurance, even compared with their counterparts in married different-sex couples (eFigure 1 in Supplement 1).

The Table presents regression results that support our trend illustrations in Figure 1. We found that, on average, individuals in same-sex couples were 1.7 to 2.5 percentage points more likely to be insured than their counterparts in different-sex couples. In baseline models, individuals in same-sex couples were 1.9 percentage points (CI, 0.009-0.029) more likely to be inured relative to those in different-sex couples, while in our most conservative models, adjusted for covariates and with state and year fixed effects, they were 1.7 percentage points (CI, 0.007-0.027) more likely to be insured. In final models that fully interact marital status, sex, and same-sex couple status, we found that these gains almost exclusively accrued to individuals in married couples, with both women and men in married same-sex couples approximately 4 percentage points more likely to be insured.

**Table.** Probability of Health Insurance Coverage Among Same-Sex Couples Relative to Different-Sex Couples^a^

Model and type of couple	Probability of insurance coverage, percentage point (95% CI)	*P* value
All same-sex couples		
Baseline	0.019 (0.009 to 0.029)	.001
Adjusted for covariates^b^	0.025 (0.014 to 0.037)	.001
With year and state fixed effects	0.017 (0.007 to 0.027)	.001
Fully interacted model^c^		
Married same-sex couple	0.041 (0.032 to 0.049)	<.001
Married male same-sex couple	0.043 (0.035 to 0.051)	<.001
Unmarried male same-sex couple	0.007 (−0.008 to 0.021)	.36
Unmarried female same-sex couple	−0.004 (−0.016 to 0.008)	.51

^a^ Data are from the American Community Survey, 2013 to 2022, and the sample is all cohabiting couples aged 18 to 64 years. The mean health insurance coverage rate is 0.889 (88% insured rate).

^b^ Adjusted for race, ethnicity, age, marital status, educational attainment, income, and employment status.

^c^ Reference group is an individual in a different-sex couple.

**Figure 2.**
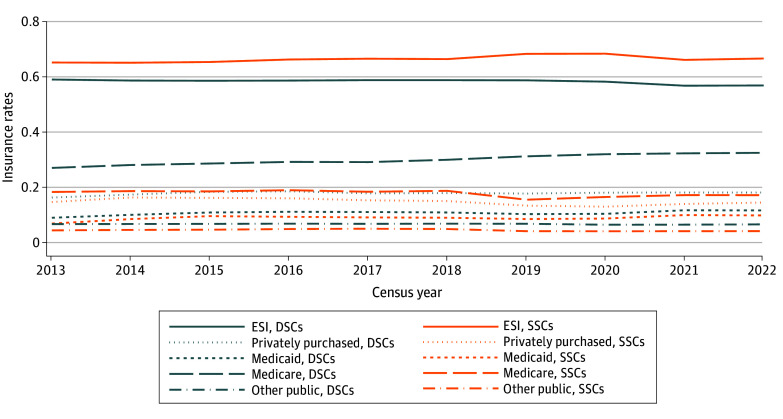
Insurance Rates Among Same-Sex Couples (SSCs) and Different-Sex Couples (DSCs), by Insurance Type, 2013 to 2022 Other public insurance includes coverage through US Department of Veterans Affairs, TriCare, and the Indian Health Service. This analysis includes adults aged 65 years and older. ESI indicates employer-sponsored insurance.

**Figure 3.**
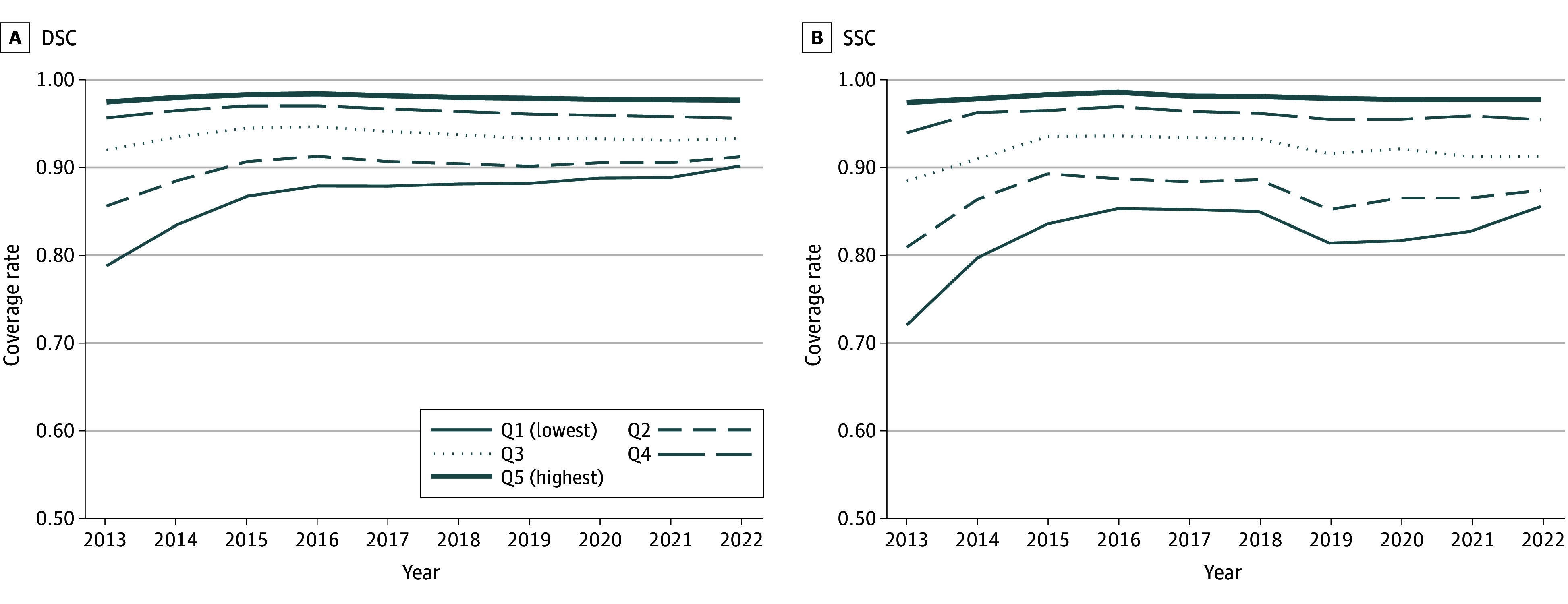
Insurance Rates Among Same-Sex Couples (SSCs) or Different-Sex Couples (DSCs), by Income Quintile (Q), 2013 to 2022

eFigure 2 in Supplement 1 and Figures 2 and 3 present the results of our secondary analyses. eFigure 2 in Supplement 1 presents trends over time for same-sex and different-sex couples in all 50 states and the District of Columbia. States in which coverage for same-sex couples persistently fell below that for different-sex couples were characterized by several key similarities: they were in the South and Midwest census regions, and they were disproportionately states that had not yet expanded Medicaid (Alabama, Georgia, Kansas, Mississippi, South Carolina, Tennessee, and Wyoming). This aligned with previous research showing that state^3^ and regional^9^ variation in insurance rates, especially public insurance rates, may be masked by broad improvements in national trends.

Figure 2 presents disaggregated insurance rates across sources of coverage. We found, importantly, in this analysis that the premium to coverage that some same-sex couples enjoy can be almost completely explained as a premium to employer-sponsored insurance. Same-sex couples are persistently covered at lower rates for all other types of coverage, including (as discussed previously), large public health insurance programs like Medicaid. For example, SSCs were insured by employers at a rate of 66.4% (12 852 of 19 358) in 2016, while DSCs were insured by employers at a rate of 58.8% (1 120 536 of 1 905 281). On the other hand, in that same year, SSCs reported any public insurance at 29.0% (5 606 of 19 358) while DSCs reported public insurance rates at 39.8% (757 856 of 1 905 281). We removed previous sample age restrictions in this analysis to include same-sex and different-sex couples aged 65 years and older. We found that coverage rates for Medicare were persistently lower for same-sex couples, likely due to the relatively lower average age of the sample of same-sex couples.

Finally, Figure 3 presents coverage rates for same-sex and different-sex couples across the income distribution. There was virtually no difference between same-sex and different-sex couples in rates of coverage among the first and second quintiles, but there was significant variation at the middle and bottom of the income distribution.

## Discussion

In this cross-sectional study, we documented the gains in health insurance status among individuals in same-sex couples in the past decade in the US. These gains, mostly realized by individuals in married same-sex couples, likely due to marriage legalization, have reversed one of the defining health disparities for SGM adults of the late 20th century. However, these gains have not been equally distributed. Instead, recent gains in insurance coverage among same-sex couples have largely been concentrated among key groups: male couples, married couples, those insured by plans offered by their employers, those in states that have expanded Medicaid, and those at the top of the income distribution. What we found aligns with and expands on previous research^1^: even though the gains to marriage accrued almost exclusively to coupled individuals, even among same-sex couples, there was significant variation in coverage across the income distribution. This suggests that human capital may be underlying our main results documenting the marital benefit: marriage is positively associated with socioeconomic status, which is associated with health insurance coverage.

### Limitations and Strengths

This study is not without limitations. It is descriptive in nature; thus, we cannot parse the specific causal mechanisms (eg, nationwide legalization of same sex marriage, the ACA, or other policies) underlying these improvements. We are also unable to directly observe sexual orientation, which prevents us from exploring trends for the largest SGM group: bisexual individuals. We are also unable to observe those whose SGM statuses overlap, an important consideration given recent state and federal legislative threats to coverage of gender-affirming care in public health insurance programs like Medicaid.

Despite these limitations, the current study has several strengths worth highlighting. We used the largest publicly available sample of SGM adults in the US. While other data products allow for the direct observation of sexual orientation, they do not lend a sample size large enough to examine the variety of stratifications we examine here. Furthermore, we extended previous literature documenting how nationwide legalization of same-sex marriage and key provisions of the ACA have narrowed the gap in insurance coverage for SGM adults^1,2,3,4^ to illustrate that recent gains in coverage do not hold for all SGM adults.

## Conclusions

In this cross-sectional study, we found that recent gains in health insurance status amoung same-sex couples were not equally distributed. They were instead mostly realized by male couples, married couples, those insured by plans offered by their employers, those in states that have expanded Medicaid, and those at the top of the income distribution. What we have documented in this study is especially important given the increasing threats that SGM populations face.^14^ The collection of sexual orientation and gender identity data are at risk, and protections for SGM individuals are likely to be erased.^14^ With reversals of SGM protections, it is thus plausible that the gains we observed may reverse. Furthermore, for same-sex couples who did not observe these gains, gaps in insurance coverage may increase. With that in mind, efforts to retain existing SGM protections must be expanded to ensure that these gains are distributed across the income distribution, to unmarried couples, and to those living in states that have not yet expanded access to public health insurance.

## Notes

Supplement 1. **eTable.** Weighted Sample Demographics **eFigure 1**. Insurance Rates Among Couples 18-64 in the US **eFigure 2**. Insurance Rates by Couple Type and State

Supplement 2.
Data Sharing Statement
