# Supplementary material for: Health Insurance Coverage Among Same-Sex vs Different-Sex Couples
Source: JAMA Netw Open. 2025 Sep 19;8(9):e2532844. doi: 10.1001/jamanetworkopen.2025.32844 (PMC12449720; doi:10.1001/jamanetworkopen.2025.32844)
Supplement: Supplement 1. — eTable. Weighted Sample Demographics eFigure 1. Insurance Rates Among Couples 18-64 in the US eFigure 2. Insurance Rates by Couple Type and State [file jamanetwopen-e2532844-s001.pdf]

Supplemental Online Content

Harrell BJ, Jones NE, Miller GH. Health insurance coverage among same-sex vs different-sex couples. *JAMA Netw Open.* 2025;8(9):e2532844.
doi:10.1001/jamanetworkopen.2025.32844

eTable. Weighted Sample Demographics

eFigure 1. Insurance Rates Among Couples 18-64 in the US

eFigure 2. Insurance Rates by Couple Type and State

This supplemental material has been provided by the authors to give readers additional information about their work.

© 2025 Harrell BJ et al. *JAMA Network Open*.

eTable. Weighted Sample Demographics				
	**Different-Sex Couple (n=20,712,935)**	**Same-Sex Couple (n=225,579)**	**Total (n=20,938,414)**	**Difference**
Any Health Insurance Coverage	18,382,787 (89%)	204,471 (91%)	18,587,258 (89%)	<0.001
Public Health Insurance Coverage	3,422,683 (17%)	30,808 (14%)	3,453,491 (16%)	<0.001
Private Health Insurance Coverage	14,086,799 (68%)	161,284 (71%)	14,248,083 (68%)	<0.001
Age	45.59 (11.86)	43.65 (11.87)	45.57 (11.86)	<0.001
White	16,095,800 (78%)	180,908 (80%)	16,276,708 (78%)	<0.001
Black	1,801,149 (9%)	14,240 (6%)	1,815,389 (9%)	<0.001
Hispanic	2,585,577 (12%)	29,634 (13%)	2,615,211 (12%)	<0.001
Asian	1,153,836 (6%)	8,864 (4%)	1,162,700 (6%)	<0.001
Other Race	1,662,050 (8%)	21,567 (10%)	1,683,617 (8%)	<0.001
Female	11,024,010 (53%)	115,496 (51%)	11,139,506 (53%)	<0.001
Married	13,897,942 (67%)	90,472 (40%)	13,988,414 (67%)	<0.001
B.A. or Higher	7,418,968 (36%)	111,228 (49%)	(7,530,196) 36%	<0.001
Household Income	114,162.8 (100,275.7)	129,666.1 (106,013.4)	99,488.05	<0.001
Employed	76.00	81.00	76.00	<0.001
Source: American Community Survey (ACS), 2008-2022; Sample is all cohabiting couples aged 18-64				

© 2025 Harrell BJ et al. *JAMA Network Open*.

eFigure 1

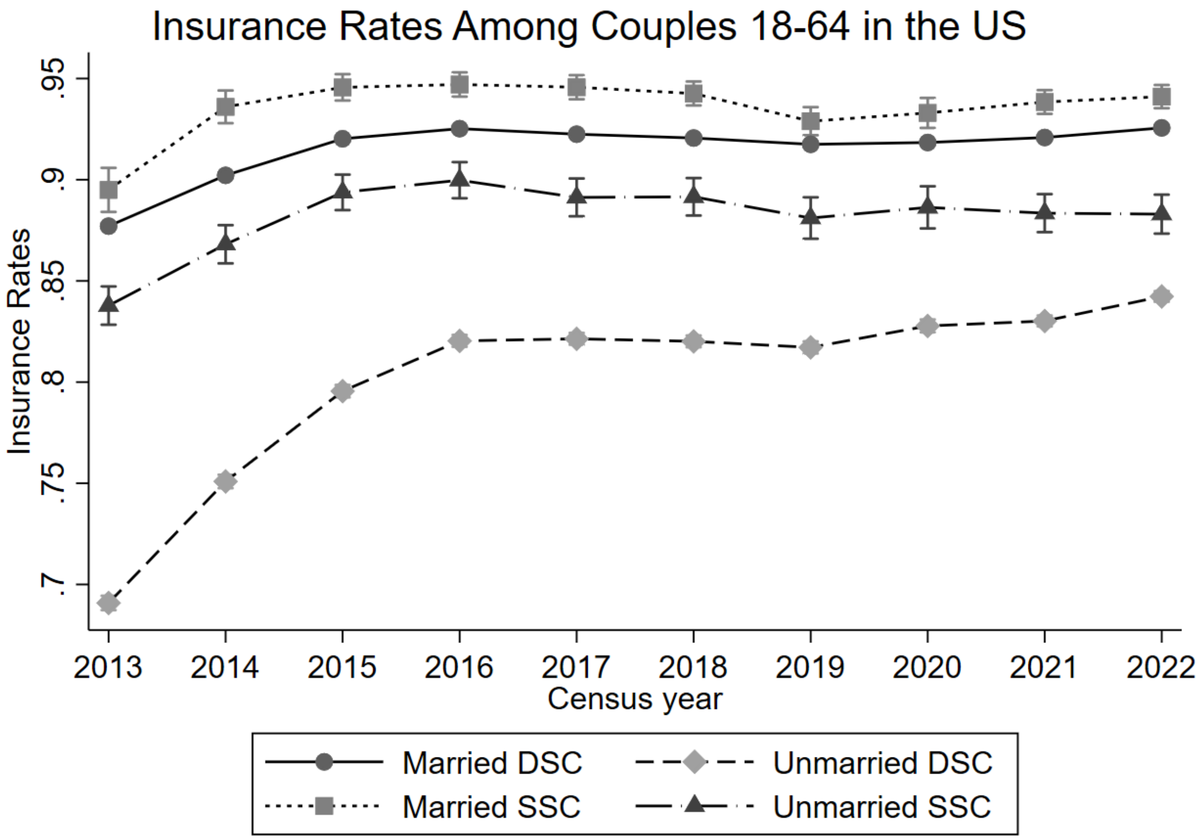

© 2025 Harrell BJ et al. *JAMA Network Open*.

eFigure 2

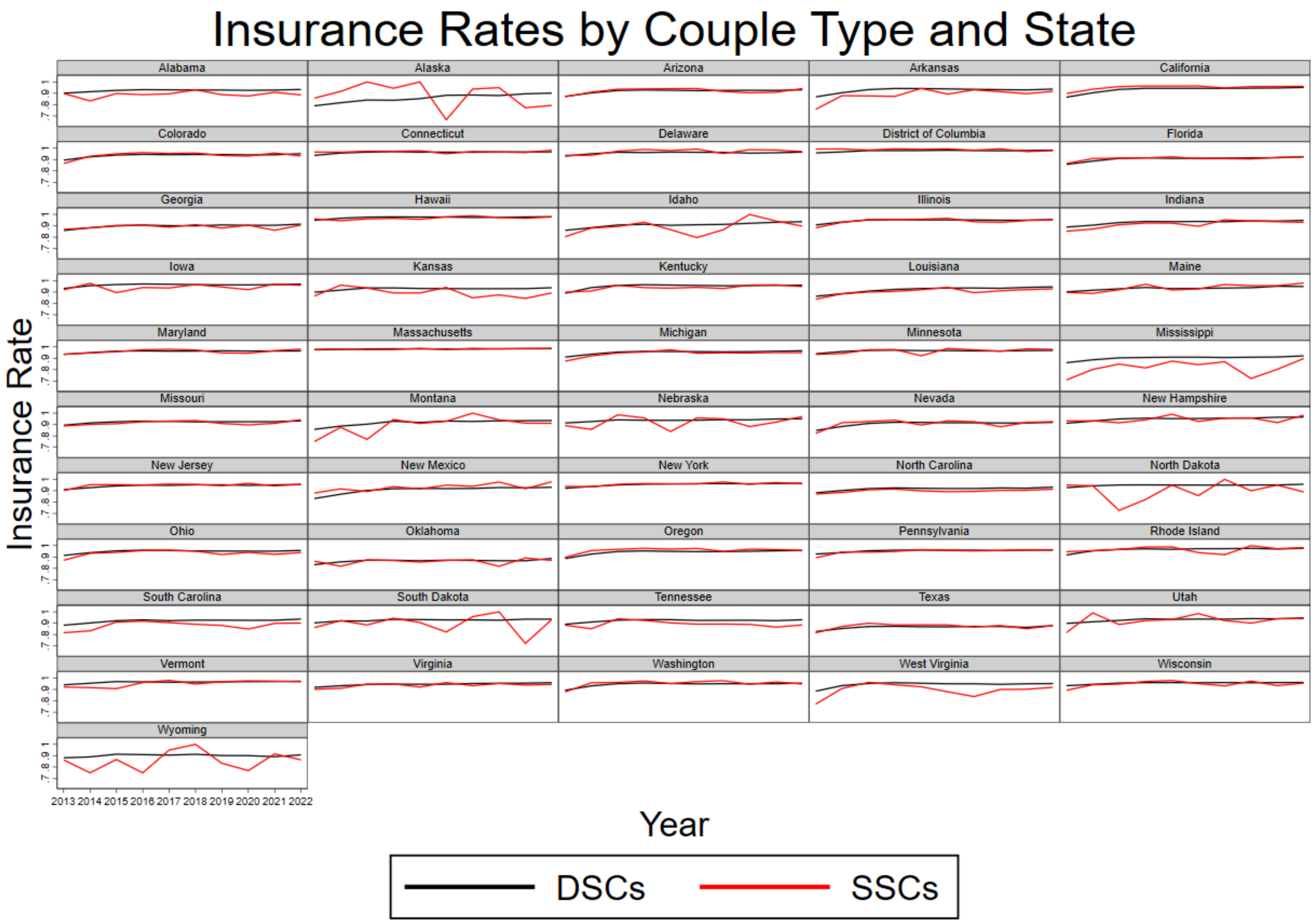

© 2025 Harrell BJ et al. *JAMA Network Open*.
