# Supplementary material for: Health Insurance Coverage Among Same-Sex vs Different-Sex Couples
Source: JAMA Netw Open. 2025 Sep 19;8(9):e2532844. doi: 10.1001/jamanetworkopen.2025.32844 (PMC12449720; doi:10.1001/jamanetworkopen.2025.32844)
Supplement: Supplement 2. — Data Sharing Statement [file jamanetwopen-e2532844-s002.pdf]

Data Sharing Statement

Harrell. Health Insurance Coverage Among Same-Sex vs Different-Sex Couples. *JAMA Netw Open*. Published September 19, 2025. doi:10.1001/jamanetworkopen.2025.32844

Data
Data available: No

Additional Information
Explanation for why data not available: Data is available through Steven Ruggles, Sarah Flood, Matthew Sobek, Daniel Backman, Grace Cooper, Julia A. Rivera Drew, Stephanie Richards, Renae Rodgers, Jonathan Schroeder, and Kari C.W. Williams. IPUMS USA: Version 16.0 [dataset]. Minneapolis, MN: IPUMS, 2025. https://doi.org/10.18128/D010.V16.0
